# Isolation and Pharmacological Characterisation of Pre-Synaptic Neurotoxins from Thai and Javanese Russell’s Viper (*Daboia siamensis*) Venoms

**DOI:** 10.3390/toxins16090405

**Published:** 2024-09-19

**Authors:** Mimi Lay, Wayne C. Hodgson

**Affiliations:** Monash Venom Group, Department of Pharmacology, Biomedical Discovery Institute, Monash University, Clayton, VIC 3800, Australia; mimi.lay@monash.edu

**Keywords:** snake venom, neurotoxin, Russell’s viper, neuromuscular junction, phospholipase A_2_

## Abstract

The widespread geographical distribution of Russell’s vipers (*Daboia* spp.) is associated with marked variations in the clinical outcomes of envenoming by species from different countries. This is likely to be due to differences in the quantity and potency of key toxins and, potentially, the presence or absence of some toxins in venoms across the geographical spectrum. In this study, we aimed to isolate and pharmacologically characterise the major neurotoxic components of *D. siamensis* venoms from Thailand and Java (Indonesia) and explore the efficacy of antivenom and a PLA_2_ inhibitor, Varespladib, against the neuromuscular activity. These data will provide insights into the link between venom components and likely clinical outcomes, as well as potential treatment strategies. Venoms were fractionated using RP-HPLC and the in vitro activity of isolated toxins assessed using the chick biventer cervicis nerve-muscle preparation. Two major PLA_2_ fractions (i.e., fractions 8 and 10) were isolated from each venom. Fraction 8 from both venoms produced pre-synaptic neurotoxicity and myotoxicity, whereas fraction 10 from both venoms was weakly neurotoxic. The removal of the two fractions from each venom abolished the in vitro neurotoxicity, and partially abolished myotoxicity, of the whole venom. A combination of the two fractions from each venom produced neurotoxic activity that was equivalent to the respective whole venom (10 µg/mL), but the myotoxic effects were not additive. The in vitro neurotoxicity of fraction 8 (100 nM) from each venom was prevented by the pre-administration of Thai Russell’s viper monovalent antivenom (2× recommended concentration) or preincubation with Varespladib (100 nM). Additionally, the neurotoxicity produced by a combination of the two fractions was partially reversed by the addition of Varespladib (100–300 nM) 60 min after the fractions. The present study demonstrates that the in vitro skeletal muscle effects of Thai and Javanese *D. siamensis* venoms are primarily due to key PLA_2_ toxins in each venom.

## 1. Introduction

*Daboia siamensis*, commonly known as Russell’s vipers, have significant geographical separation across the Asiatic mainland. Coupled with evolutionary processes, this has led to substantial variations in venom composition both between and within species, resulting in pronounced differences in the symptoms observed in envenomed patients [1]. However, all *Daboia* venoms contain a high percentage of kunitz-type serine protease inhibitors (KSPI), phospholipase A_2_ (PLA_2_), snake venom serine proteases (SVSPs), and snake venom metalloproteases (SVMPs) [2,3,4,5]. *Daboia siamensis* venoms exhibit considerable toxicity resulting from the cumulative or synergistic effects of multiple toxins [6]. There is considerable geographical variation in the clinical outcomes of *D. siamensis* envenoming. For example, in Thailand, while only 2% of snakebite fatalities result from *D. siamensis* [7], envenoming may result in intravascular haemolysis and reduced blood coagulability. However, generalised capillary permeability and shock are generally absent following envenoming by Thai *D. siamensis* [8,9]. In addition, patients have a high risk of developing rhabdomyolysis if left untreated [10]. In contrast, while there are low numbers of snakebite related incidents and deaths registered in Indonesia, Indonesian *D. siamensis* envenoming may result in reduced blood coagulability, mild blistering necrosis and, less frequently, very mild neurotoxicity, i.e., ptosis [11,12].

PLA_2_ toxins are among the most abundant components of viperid venoms and exhibit a wide range of pharmacological activities, including neurotoxicity, myotoxicity, haemostatic disturbances, cardiotoxicity, and hypotension [13]. Similarities in the high content of PLA_2_ toxins have been noted between *D. siamensis* species in Myanmar, China, Taiwan, Thailand, and Indonesia [4,14,15,16]. Different isoforms of toxins in the venom would give rise to different types of toxicity depending on the toxin target and could be responsible for varied clinical effects following envenoming [17]. Notably, neuromuscular inhibition, which is typically caused by PLA_2_ neurotoxins, is largely absent following *D. siamensis* envenoming [5,18]. Our recent investigation into the neurotoxic effects of Thai or Javanese *D. siamensis* venoms revealed that neurotoxicity might be species-dependent, despite the presence of neurotoxic PLA_2_ components [19]. Previous studies focused on the activity of whole venoms. Consequently, further investigation was required to isolate these neurotoxins and describe their in vitro pharmacological properties.

Most viper venoms may contain one or both types of PLA_2_ toxins, commonly referred to as the ‘Asp49’ and ‘Lys49’ variants. The Lys49 types are homologous PLA_2_-like proteins because they are enzymatically inactive compared to the Asp49 counterparts [13,20,21,22,23]. For example, β-neurotoxins, or pre-synaptic PLA_2_ neurotoxins, which inhibit the release of acetylcholine at the skeletal neuromuscular junction, can be found as a protein complex with either of the two types of PLA_2_ toxins, or function as an accessory protein in a more complex structure containing other types of protein apart from PLA_2_s [24]. There are differences in the structural composition of β-neurotoxins. For example, ammodytoxin A from *Vipera ammodytes ammodytes* venom is a PLA_2_ monomer, whereas crotoxin isolated from *Crotalus durissus terrificus* venom consists of a heterodimeric complex made up of a basic, toxic PLA_2_ (crotoxin B) and an acidic non-enzymatic protein (crotoxin A) that enhances the toxicity of crotoxin B [25,26]. Several pre-synaptic neurotoxins have been isolated from Russell’s viper venoms. However, apart from U1-viperitoxin-Dr1a from Sri-Lankan *D. russelii* venom, which has been reported to cause neurotoxicity in humans [27], other PLA_2_ neurotoxins including Daboiatoxin and potentially Russtoxin/Viperotoxin-F (isolated from Russell’s viper in Myanmar and Taiwan, respectively) have not been typically reported to cause neurotoxicity in humans [15,16,28,29].

Viperid venoms contain either single-chain β-neurotoxins or dimeric β-neurotoxins. The latter usually involves association with a chaperone (typically a PLA_2_) subunit to enhance the affinity and toxicity of a complex [25]. Recent profiling of the Thai and Indonesian *D. siamensis* venom proteomes revealed highly homologous PLA_2_ isoforms to Viperotoxin-F, a PLA_2_ complex isolated from Taiwanese *D. siamensis* venom [3,15,29]. This complex shares the biological activity of crotoxin, where its lethal potency and neuromuscular blocking effects, both in vitro or in vivo, are potentiated in the dimeric form [2,15,16]. Other studies on whole *D. siamensis* venom have demonstrated high lethal potency, incoagulable blood and varying degrees of capillary leakage and renal failure in in vivo mice [4,30,31,32]. These effects were attributed to the high PLA_2_, SVSP, and SVMP contents [30,31,32].

The lack of clinically evident neurotoxicity following envenoming by Thai and Javanese *D. siamensis* is interesting from a biological perspective. Isolated toxins may exert specific toxicity experimentally and demonstrate different activity than whole venom in different models. For example, crotoxin is functionally a neurotoxin but may also cause systemic and local myotoxicity in vivo [33]. Additionally, haemorrhagic complex I, isolated from *D. russelii* venom functions to induce haemorrhage in mice at the site of injection, while its individual components consist of a non-haemorrhagic PLA_2_ and a neurotoxic non-enzymatic peptide [34].

Our recent characterisation of Javanese and Thai *D. siamensis* venoms found potent pre-synaptic neurotoxic effects [19], despite the reported absence of neurotoxicity in envenomed patients [35]. The specific contribution of these PLA_2_ toxins to the overall venom activity remains relatively unexplored. Consequently, there is a gap in our knowledge of how these toxins influence the outcomes of envenoming and how they respond to therapeutic intervention. This prompted us to study the key PLA_2_ toxins from these venoms to provide further insights into the properties of these toxins.

## 2. Results

### 2.1. Whole D. siamensis Venom In Vitro Neurotoxicity and Myotoxicity

**Figure 1:**
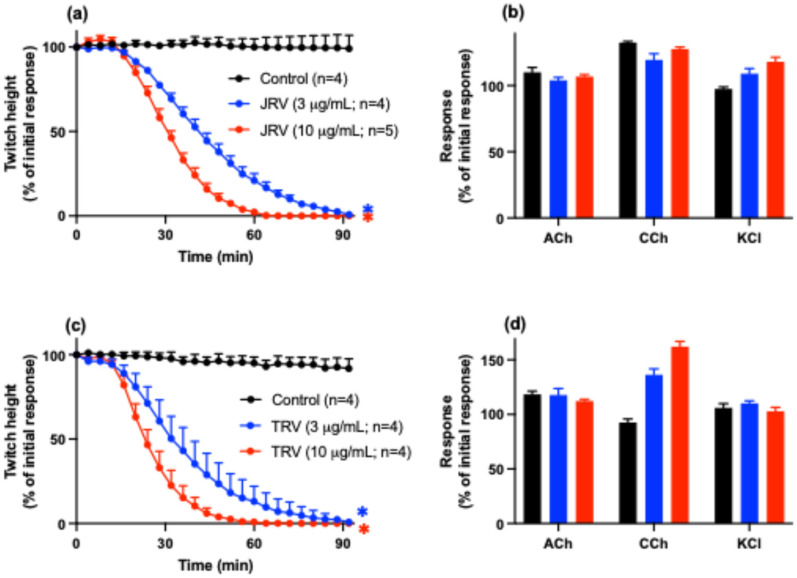

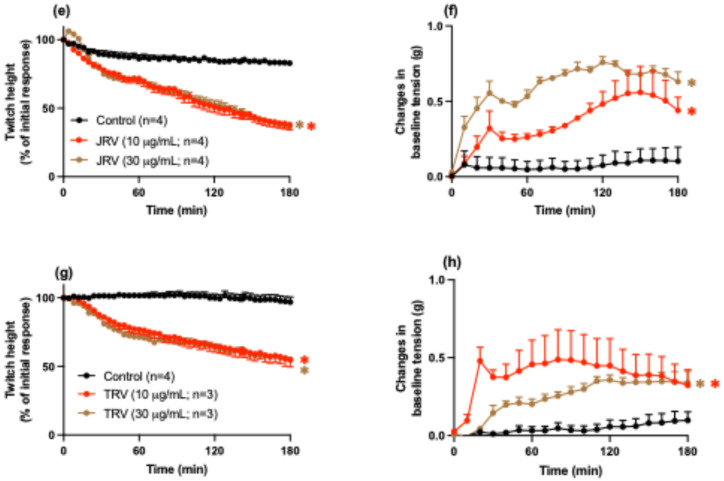
Effect of Javanese (JRV) or Thai (TRV) *D. siamensis* venoms (3–10 µg/mL) on (**a**,**c**) indirect twitches and (**b**,**d**) contractile responses to acetylcholine (ACh), carbachol (CCh), and potassium chloride (KCl) in the chick biventer cervicis nerve-muscle preparation. Effect of Javanese (JRV) or Thai (TRV) *D. siamensis* venoms (10–30 µg/mL) on (**e**,**g**) direct twitches or (**f**,**h**) baseline tension in the chick biventer cervicis nerve-muscle preparation. Data presented as mean ± SEM; * *p* < 0.05, significantly different from vehicle control (Control); one-way ANOVA followed by Bonferroni multiple comparison post hoc test, n = 3–5.

Both Javanese (JRV; Figure 1a) and Thai (TRV; Figure 1c) *D. siamensis* venoms (3 and 10 µg/mL) abolished indirect twitches of the chick biventer cervicis nerve-muscle preparation. Neither venom significantly inhibited responses to exogenous acetylcholine (ACh), carbachol (CCh), or potassium chloride (KCl) (Figure 1b,d), indicating a pre-synaptic mode of action. The times to reach 90% inhibition (t_90_) of indirect twitches for the Javanese venom were 71 ± 3 min and 49 ± 3 min for 3 µg/mL and 10 µg/mL, respectively. For the Thai venom, the t_90_ values were 57 ± 10 min at 3 µg/mL, and 37 ± 3 min at 10 µg/mL.

Additionally, both Javanese (JRV, Figure 1e,f) and Thai (TRV; Figure 1g,h) *D. siamensis* venoms (10–30 µg/mL) inhibited direct twitches and caused an increase in baseline tension indicative of myotoxicity.

### 2.2. Isolation of Toxins

#### 2.2.1. Fractionation of *D. siamensis* Venom via Reverse-Phase HPLC

**Figure 2:**
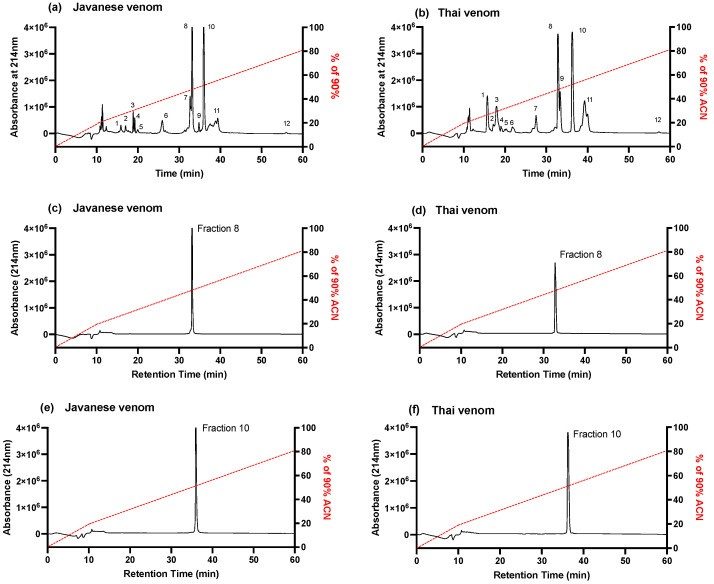
HPLC chromatograms of (**a**) Javanese and (**b**) Thai *D. siamensis* venoms using a Jupiter C18 semi-preparative column. Chromatograms of isolated fractions 8 and 10 from (**c**,**e**) Javanese and (**d**,**f**) Thai *D. siamensis* venoms.

The RP-HPLC fractionation of Javanese and Thai *D. siamensis* venoms, using a Jupiter C18 semi-preparative column, yielded a number of major and minor peaks from both venoms (labelled 1–12; Figure 2a,b). Fractions 8 and 10 from the Javanese *D. siamensis* venom were large peaks with percentages of 20% (34 min elution time; Figure 2c) and 23% (36 min elution time; Figure 2e) of the whole venom (Figure 2a). Fractions 8 and 10 from the Thai venom eluted at the same time points (Figure 2d,f) and constituted 20% and 23%, respectively, of the whole venom (Figure 2b). As fractions 8 and 10, together, constituted >40% of the whole venoms, and eluted at similar time points to neurotoxins previously isolated from Sri Lankan *D. russelii* venom [21], they were examined for activity using the chick biventer cervicis nerve-muscle preparation.

#### 2.2.2. Intact Protein Analysis

**Figure 3:**
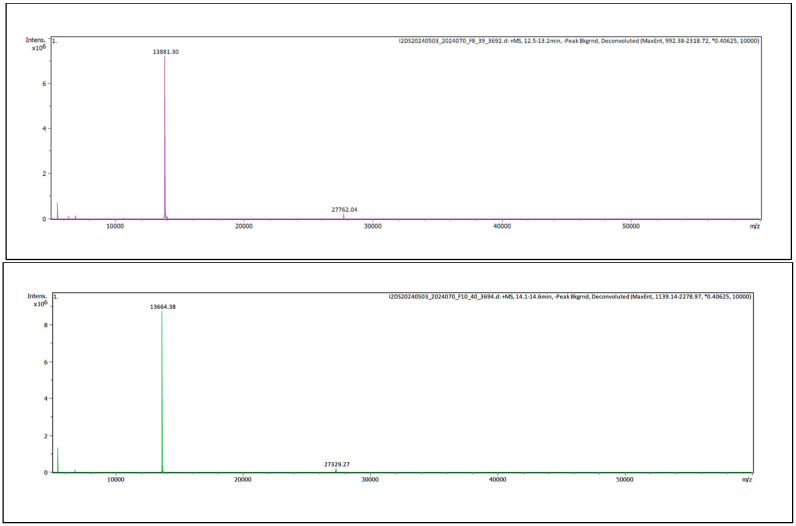
Intact mass protein analysis LC-ESI-MS chromatogram of (**upper panel**) fraction 8 and (**lower panel**) fraction 10 from Javanese *D. siamensis* venom.

Analysis of fractions 8 and 10 from Javanese *D. siamensis* venom by mass spectrometry indicated masses of 13,881 Da and 13,664 Da, respectively (Figure 3).

**Figure 4:**
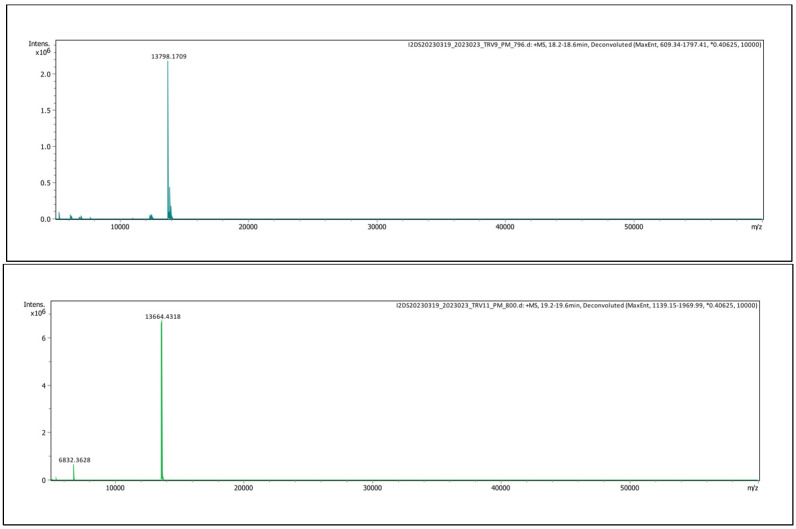
Intact mass protein analysis LC-ESI-MS chromatogram of (**upper panel**) fraction 8 and (**lower panel**) fraction 10 from Thai *D. siamensis* venom.

Analysis of Thai *D. siamensis* venom by mass spectrometry indicated masses of 13,798 Da for fraction 8 and 13,664 Da for fraction 10, respectively (Figure 4).

### 2.3. In Vitro Neurotoxicity and Myotoxicity

#### 2.3.1. Javanese *D. siamensis* Venom Fractions

**Figure 5:**
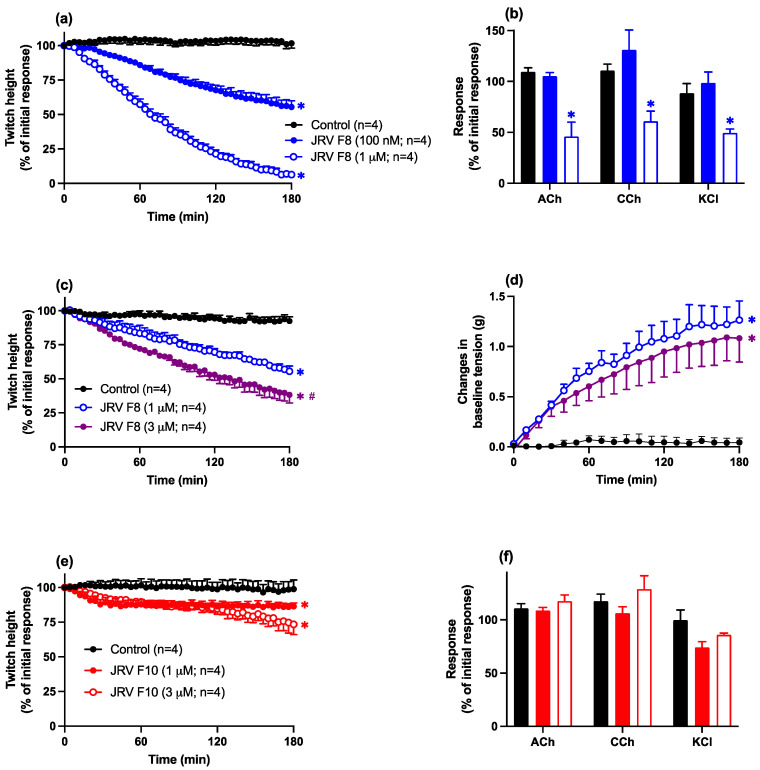
The effects of Javanese *D. siamensis* fraction 8 (100 nM–3 µM) on (**a**) indirect twitches or (**b**) contractile responses to exogenous ACh, CCh, or KCl or (**c**) direct twitches or (**d**) baseline tension of the chick biventer cervicis nerve-muscle preparation. Effects of Javanese *D. siamensis* fraction 10 (1–3 µM) on (**e**) indirect twitches or (**f**) contractile responses to exogenous ACh, CCh, or KCl in the chick biventer cervicis nerve-muscle preparation. Data presented as the mean ± SEM, * *p* < 0.05, significantly different from vehicle control (Control) at 180 min; # *p* < 0.05, significantly different from same fraction at a lower concentration; one-way ANOVA followed by Bonferroni multiple comparison post hoc test; * *p* < 0.05, significantly different from pre-venom response; student’s paired *t*-test, n = 4.

Fraction 8 (100 nM–1 µM) produced concentration-dependent inhibition of indirect twitches in the chick biventer preparation (Figure 5a). A lack of an inhibitory effect of fraction 8 (100 nM) on contractile responses to exogenous ACh, CCh, and KCl indicated a pre-synaptic neurotoxic mode of action (Figure 5b). The time to reach 90% inhibition (i.e., t_90_) of indirect twitches for fraction 8 (1 µM) was 157 ± 10 min.

At a higher concentration (i.e., 1 µM), a non-selective inhibitory effect on contractile responses to exogenous agonists was observed, indicative of myotoxicity (Figure 5b). This myotoxic effect was confirmed by an inhibitory effect of fraction 8 (1–3 µM) on direct twitches of the chick biventer (Figure 5c) with a corresponding increase in baseline tension (Figure 5d).

Fraction 10 (1–3 µM; Figure 5e,f) produced a much weaker inhibition of indirect twitches, with no inhibitory effect on contractile responses to exogenous agonists, indicating a weak pre-synaptic neurotoxic effect.

#### 2.3.2. Thai *D. siamensis* Venom Fractions

**Figure 6:**
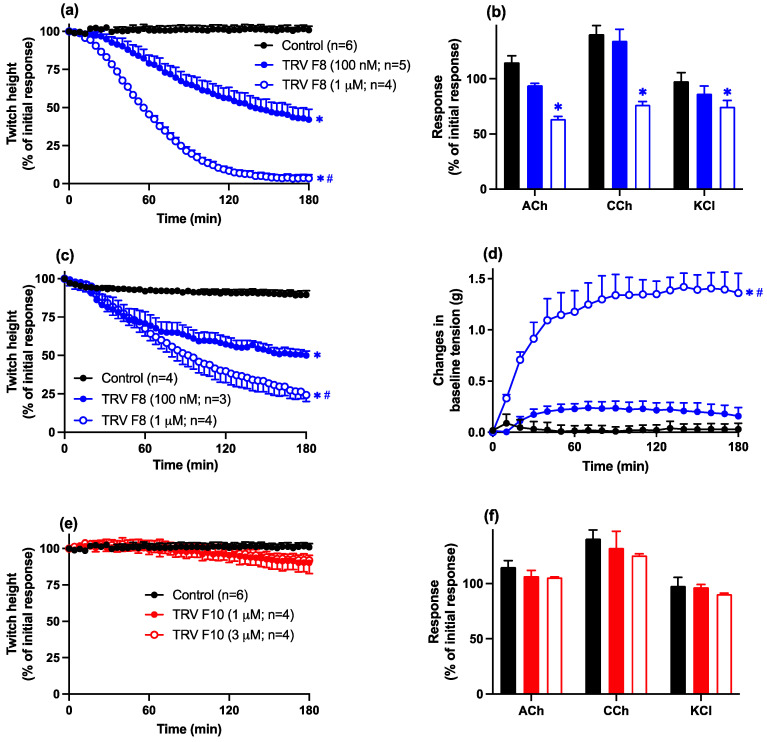
The effects of Thai *D. siamensis* fraction 8 (100 nM–1 µM) on (**a**) indirect twitches or (**b**) contractile responses to exogenous ACh, CCh, or KCl or (**c**) direct twitches or (**d**) baseline tension. Effects of fraction 10 (1–3 µM) on (**e**) indirect twitches or (**f**) contractile responses to exogenous ACh, CCh, or KCl in the chick biventer cervicis nerve-muscle preparation. Data presented as the mean ± SEM, * *p* < 0.05, significantly different from vehicle control (Control) at 180 min; # *p* < 0.05, significantly different from the same fraction at a lower concentration; one-way ANOVA followed by Bonferroni multiple comparison post hoc test; * *p* < 0.05, significantly different from pre-venom response; student’s paired *t*-test, n = 3–6.

Fraction 8 (100 nM–1 µM) produced concentration-dependent inhibition of indirect twitches in the chick biventer preparation (Figure 6a). A lack of an inhibitory effect of fraction 8 (100 nM) on contractile responses to exogenous ACh, CCh, and KCl indicated a pre-synaptic neurotoxic mode of action (Figure 6b). The time to reach 90% inhibition (i.e., t_90_) of indirect twitches for fraction 8 (1 µM) was 117 ± 8 min.

At a higher concentration (i.e., 1 µM), a non-selective inhibitory effect on contractile responses to exogenous agonists was observed, indicative of myotoxicity (Figure 6b). This myotoxic effect was confirmed by an inhibitory effect of fraction 8 (100 nM–1 µM) on direct twitches of the chick biventer (Figure 6c) with a corresponding increase in baseline tension (Figure 6d).

Fraction 10 (1–3 µM; Figure 6e,f) had no significant inhibitory effect on indirect twitches, or contractile responses to exogenous agonists, in the chick biventer preparation, indicating the apparent absence of neurotoxicity or myotoxicity.

#### 2.3.3. Removal, or Combined Activity, of Fractions 8 and 10 from Javanese and Thai *D. siamensis* Venoms

**Figure 7:**
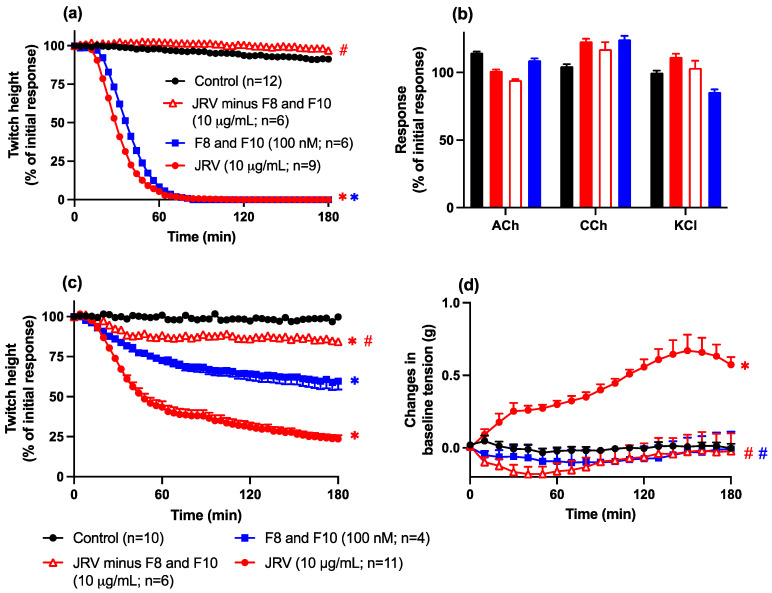
Comparison of the effect of whole Javanese *D. siamensis* venom (10 µg/mL), venom devoid of fractions 8 and 10 (10 µg/mL), and fractions 8 and 10 combined (at a 1:1 ratio; 100 nM) on (**a**) indirect twitches, (**b**) contractile responses to exogenous ACh, CCh, or KCl, (**c**) direct twitches, or (**d**) baseline tension of the chick biventer cervicis nerve-muscle preparation. Data presented as the mean ± SEM, * *p* < 0.05, significantly different from vehicle control (Control) at 180 min; # *p* < 0.05, significantly different from whole venom at 180 min; one-way ANOVA followed by Bonferroni multiple comparison post hoc test, n = 4–12.

The removal of fractions 8 and 10 from Javanese *D. siamensis* venom resulted in the loss of neurotoxicity of the remaining venom (10 μg/mL; Figure 7a,b). However, some residual myotoxic activity (i.e., small but significant inhibition of direct twitches) was observed for the venom in the absence of fractions 8 and 10, although the increase in baseline tension produced by the intact whole venom was no longer observed.

The combination of fractions 8 and 10 from Javanese *D. siamensis* venom (100 nM) produced pre-synaptic neurotoxicity (i.e., inhibition of indirect twitches), which was not statistically different from neurotoxicity produced by the whole intact venom (10 µg/mL; Figure 7a,b). The combination of fractions 8 and 10 (100 nM) produced a myotoxic effect (i.e., inhibition of direct twitches) that was significantly greater than venom devoid of fractions 8 and 10, but not as marked as the whole intact venom (Figure 7c). This difference was mirrored by the lack of an increase in baseline tension of the combination of fractions 8 and 10, which was the same as the venom devoid of fractions 8 and 10, whereas the intact whole venom induced an increase in baseline tension (Figure 7d).

**Figure 8:**
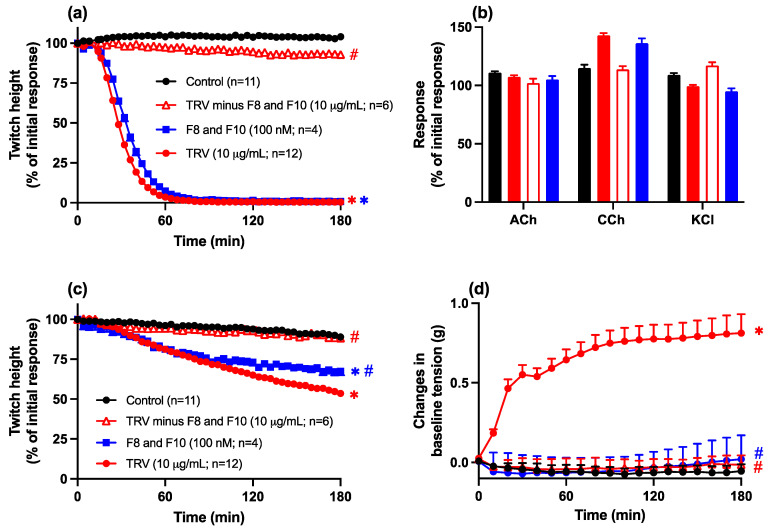
Comparison of the effect of whole Thai *D. siamensis* venom (10 µg/mL), venom devoid of fractions 8 and 10 (10 µg/mL), and fractions 8 and 10 combined (at a 1:1 ratio; 100 nM) on (**a**) indirect twitches, (**b**) contractile responses to exogenous ACh, CCh, or KCl, (**c**) direct twitches, or (**d**) baseline tension of the chick biventer cervicis nerve-muscle preparation. Data presented as the mean ± SEM, * *p* < 0.05, significantly different from vehicle control (Control) at 180 min; # *p* < 0.05, significantly different from whole venom at 180 min; one-way ANOVA followed by Bonferroni multiple comparison post hoc test, n = 4–12.

The removal of fractions 8 and 10 from Thai *D. siamensis* venom (Figure 8) also resulted in the loss of neurotoxicity (Figure 8a,b) and myotoxicity (Figure 8c,d) of the remaining venom, as indicated by the absence of inhibition of indirect twitches (Figure 8a), contractile responses to exogenous agonists (Figure 8b), and direct twitches (Figure 8c), as well as the lack of effect on baseline tension (Figure 8d). The combination of fractions 8 and 10 (100 nM) also inhibited direct twitches (Figure 8c), an effect which was slightly attenuated compared to the whole venom (10 µg/mL; Figure 8c) but did not cause an increase in baseline (Figure 8d).

### 2.4. Antivenom or Varespladib Studies

#### Neurotoxicity: Prevention Studies

**Figure 9:**
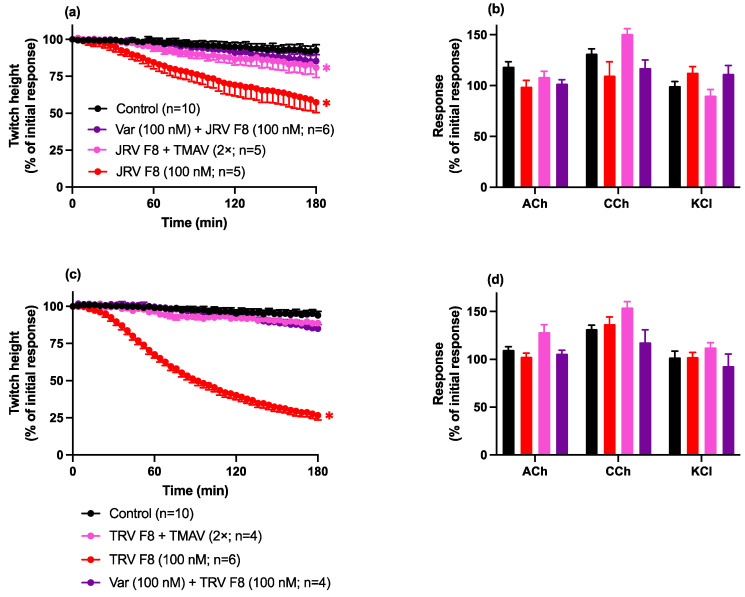
The effect of (**a**) Javanese *D. siamensis* venom fraction 8 (100 nM) or (**b**) Thai *D. siamensis* venom fraction 8 (100 nM) in the absence and presence of Varespladib (100 nM) or Thai Russell’s viper monovalent antivenom (2× recommended concentration) on (**a**,**c**) indirect twitches or (**b**,**d**) contractile responses to exogenous ACh, CCh, or KCl, in the chick biventer cervicis nerve-muscle preparation. Data presented as the mean ± SEM, * *p* < 0.05, significantly different from vehicle control (Control) at 180 min; one-way ANOVA followed by Bonferroni multiple comparison post hoc test; for agonist responses, n = 4–10.

Pre-incubation with Varespladib (100 nM), or pre addition of Thai Russell’s viper antivenom (2× recommended concentration) to the organ bath, prevented the inhibition of indirect twitches by fraction 8 from both the Javanese *D. siamensis* (Figure 9a) and Thai *D. siamensis* (Figure 9b) venoms.

**Figure 10:**
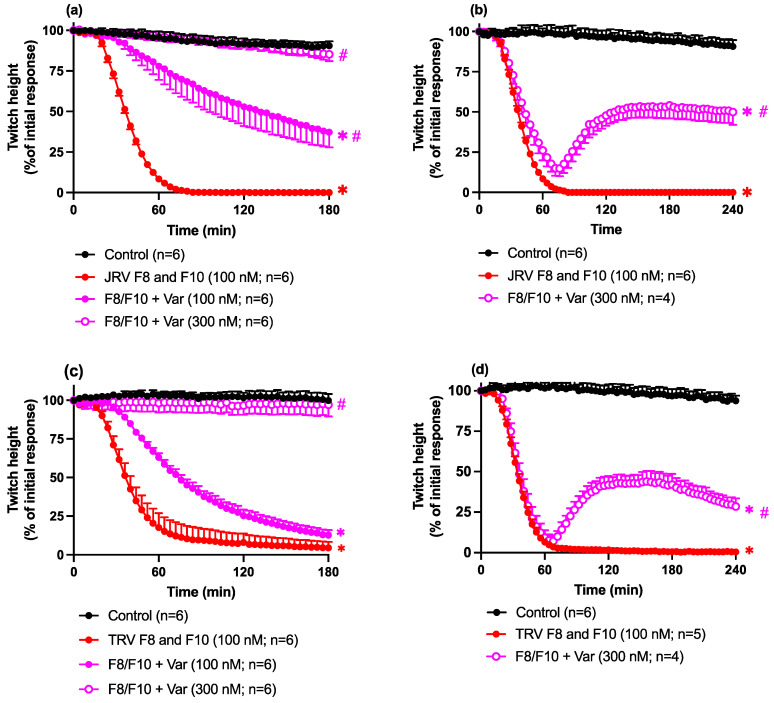
The effects of Varespladib (100–300 nM) pre-incubated with the combination of fractions 8 and 10 from (**a**) Javanese *D. siamensis* or (**c**) Thai *D. siamensis* venoms; or Varespladib (300 nM) added 60 min after the combination of fractions 8 and 10 from (**b**) Javanese *D. siamensis* or (**d**) Thai *D. siamensis* venoms in the indirectly stimulated chick biventer cervicis nerve-muscle preparation. Data presented as the mean ± SEM, * *p* < 0.05, significantly different from vehicle control (Control) at 180 (**a**,**c**) or 240 min (**b**,**d**); # *p* < 0.05, significantly different from F8/F10 alone at 180 min (**a**,**c**) or 240 min (**b**,**d**); one-way ANOVA followed by Bonferroni multiple comparison post hoc test, n = 4–6.

The pre-incubation of both toxins together with Varespladib (100–300 nM) produced concentration-dependent protection against the pre-synaptic neurotoxicity induced by the combination of fractions 8 and 10 (100 nM) from Javanese *D. siamensis* (Figure 10a) and Thai *D. siamensis* (Figure 10c) venoms. The higher concentration of Varespladib (i.e., 300 nM) caused a complete blockade of the inhibitory effect of the fractions (i.e., not significantly different from the control; Figure 10a,c). The addition of Varespladib (300 nM) 60 min after the addition of the combination of fractions 8 and 10 caused partial reversal of the pre-synaptic neurotoxicity for fractions from the Javanese *D. siamensis* (Figure 10b) and Thai *D. siamensis* (Figure 10d) venoms.

### 2.5. PLA_2_ Activity

The Javanese and Thai *D. siamensis* venoms displayed PLA_2_ activities of 1442 ± 290 µmol/min/mg (n = 3) and 2640 ± 295 µmol/min/mg (n = 3), respectively, with the activity of Thai *D. siamensis* venom being significantly higher than that of Javanese *D. siamensis* venom (* *p* < 0.05, unpaired *t*-test). Fractions 8 and 10 from the Javanese venom had activities of 1040 ± 104 µmol/min/mg (n = 5) and 181 ± 62 µmol/min/mg (n = 6), respectively. Fractions 8 and 10 from the Thai venom had activities of 4269 ± 1207 µmol/min/mg (n = 7) and 883 ± 178 µmol/min/mg (n = 7), respectively. For both venoms, fraction 8 had significantly higher PLA_2_ activity compared to fraction 10 from the same venom (* *p* < 0.05, unpaired *t*-test). Bee venom was used as a positive control and was found to have an activity of 565 ± 30 µmol/min/mg (n = 5).

## 3. Discussion

In this study, we examined the pharmacological characteristics of two major fractions isolated from Javanese *D. siamensis* and Thai *D. siamensis* venoms. Fraction 8 from both venoms exerted neurotoxic and myotoxic effects. However, this activity was largely absent in fraction 10 from each venom. Additionally, the removal of the toxins from each venom led to almost a complete loss of neurotoxicity, with some residual myotoxicity. Further, the present study confirmed the in vitro efficacy of Thai Russell’s viper monovalent antivenom to neutralise the neurotoxic activity and the ability of Varespladib to partially reverse already established pre-synaptic neurotoxicity.

Javanese and Thai *D. siamensis* venoms exhibited significant pre-synaptic neurotoxicity and mild myotoxicity in the chick biventer cervicis nerve-muscle preparation, as evidenced by the inhibition of both indirect and direct twitches, respectively. This finding aligns with proteomic analyses of *D. siamensis* venoms, which suggest a large proportion of PLA_2_ toxins in the venom [3,4,14,36,37]. Comparing the toxin profiles and potencies of different Russell’s viper venoms can provide insights into their evolutionary conservation and potential variations in their pathophysiological mechanisms. However, subtle differences in abundance and the type of PLA_2_ can alter their main toxic effects [38], such as neurotoxicity or myotoxicity. Previous research has identified and isolated PLA_2_ toxins from both Thai and Javanese *D. siamensis* venoms. Notably, RV-4 (accession ID: PA2B4_DABSI) [15,16,29] and RV-7 PLA_2_ (accession ID: PA2A7_DABSI) [15,16,29,39], originally identified in Taiwanese *D. siamensis* venom, have also been isolated from Thai *D. siamensis* venom. Additionally, two PLA_2_ toxins (accession ID: B3RFI7_DABRR and B3RFI6_DABRR) [40] have been identified in Javanese *D. siamensis* venom. These toxins, originally characterised from the venoms of different geographical populations, have been detected in multiple *Daboia* venoms. However, the specific contributions of these PLA_2_ toxins to the overall activity of the venom remain largely unexplored. The role of these PLA_2_ toxins in the envenoming process, especially their effects at the neuromuscular junction, is not well understood for Thai or Javanese *D. siamensis* venoms. Hence, our study built on these previous studies to further characterise how these PLA_2_ toxins influence the outcomes of envenoming and how they respond to therapeutic intervention. Previously, it was reported that the in vitro neurotoxicity and myotoxicity induced by the Western species, i.e., Sri Lankan *D. russelii* venom, was largely confined to two PLA_2_ toxins, subsequently named U1-viperotoxin-Dr1b and -Dr1a [27]. Similarly for the Eastern species, i.e., Taiwanese *D. siamensis*, the venom-induced pre-synaptic neurotoxicity was confined to two PLA_2_ neurotoxins, namely RV-4 and RV-7 (Viperotoxin-F). Therefore, given the similarities in the HPLC profiles of Thai, Javanese, and Taiwanese *D. siamensis* venoms [14,15,16], and the potent neurotoxicity of the Thai and Javanese venoms compared to Sri Lankan *D. russelii* venom [27], we isolated the two major fractions that were conserved across these species. The two fractions from the Javanese and Thai *D. siamensis* venoms (i.e., 8 and 10), isolated using reverse-phase HPLC, constituted approximately 40% and existed in approximately a 1:1 ratio in each venom. With a molecular mass of approximately 13 kDa, these fractions are consistent with PLA_2_ toxins [13]. This was confirmed in the present study by a PLA_2_ assay of the fractions.

In the current study, Thai *D. siamensis* venom exhibited a PLA_2_ activity of 2640 µmol/min/mg, which is consistent with previously reported PLA_2_ activity of 2000 µmol/min/mg for venom from the same species [30]. Notably, this activity is higher than that observed for Javanese *D. siamensis* venom. Proteomic profiling has shown that both Thai and Indonesian *D. siamensis* venoms contain both acidic and basic PLA_2_ enzymes, likely differing in their relative abundances or ratios [3]. Interestingly, fraction 8 from each venom displayed significantly higher PLA_2_ activity compared to fraction 10. While it is unclear whether these fractions represent strictly basic or acidic PLA_2_ toxins, they likely share characteristics with viperotoxin-F, which includes a highly toxic basic neurotoxic PLA_2_ (RV-4) and a less enzymatically active acidic component (RV-7). Indeed, these results from fractions 8 and 10 are similar to previous observations on the effects of RV-4 and RV-7. In the chick biventer preparation, RV-4, but not RV-7, blocked neuromuscular transmission [16].

We then evaluated the activity of these fractions individually. Fraction 8 from each venom caused concentration-dependent inhibition of indirect twitches, demonstrating pre-synaptic neurotoxic effects at the lower concentration and myotoxic effects at a higher concentration. Fraction 10 from Thai *D. siamensis* venom was devoid of neurotoxic effects, and fraction 10 from Javanese *D. siamensis* venom exhibited weak neurotoxic effects. Interestingly, fraction 10 from Thai *D. siamensis* venom had markedly higher PLA_2_ activity than fraction 10 from the Javanese *D. siamensis* venom although the former was devoid of toxicity while the latter displayed weak neurotoxic effects. In contrast, fraction 8 from each *D. siamensis* venom exhibited markedly higher PLA_2_ activity than fraction 10 from the corresponding venom and exerted more potent neurotoxic and myotoxic effects.

Interestingly, the neurotoxic activity of fraction 8 alone, from both venoms, was relatively mild but its efficacy increased considerably in the presence of fraction 10. This synergistic effect is characteristic of many PLA_2_ toxins, which often enhance toxicity when combined. From a molecular perspective, synergism can either exist as (1) intermolecular synergism, when two or more toxins interact with two or more biological pathways to increase toxicity, or (2) supramolecular synergism, when two or more toxins interact with the same target or associate to create a complex to increases toxicity [41]. For instance, supramolecular synergism exists in some *D. siamensis* venoms. In Taiwanese Russell’s viper venom, RV-4 exhibits weak affinity for nerve terminals, but RV-7, the other major component of the RV-4/RV-7 Viperotoxin-F complex, enhances the specific binding of RV-7 to nerve terminals while reducing non-specific binding and damage to other structures [16]. In addition, the potency and neuromuscular blocking activity of RV-4 is potentiated three-fold in the presence of RV-7 [16]. Additionally, Daboiatoxin, isolated from Myanmar *D. siamensis* venom, contains two PLA_2_ toxins that work synergistically to enhance overall toxicity [2]. These toxins were reported to have synergistic interactions as the non-toxic component seems to increase the specificity and affinity of the toxic subunit by preventing non-specific absorption [24]. Hence, it is possible that the two PLA_2_ fractions isolated from the Thai and Javanese species readily associate in solution to form a more toxic complex. Future studies should investigate the enzyme activity of *D. siamensis* PLA_2_ and their isoelectric points to better understand toxin synergism and overall toxicity. Variations in PLA_2_ enzymatic activity and isoelectric points can influence how these toxins interact with each other and with other venom components, potentially leading to enhanced toxic effects [42]. Understanding these biochemical properties will shed light on the mechanisms of toxin synergism.

Although sequencing data were absent in this study, it is likely that the neurotoxicity caused by Thai and Javanese venom is due to the previously identified Russtoxin-like components in venoms. However, the exact mode of interaction and whether the enhanced toxicity is due to the formation of a dimeric complex require further exploration. Although, the combination of fraction 8 and fraction 10, from each venom, induced potent pre-synaptic neuromuscular inhibition, in most cases, clinically evident neurotoxicity following bites by these species is extremely rare [8,35,43,44,45,46]. However, in our study, each fraction 8 isolated from the Javanese and Thai *D. siamensis* venoms was less potent compared to the pre-synaptic neurotoxins previously characterised in our laboratory [27,47]. For example, taipoxin at 2.04 µg/mL had a t_90_ value of approximately 63.5 min [47], while U1-viperotoxin-Dr1a (1 µM, equivalent to approximately 13 µg/mL) from Sri Lankan *D. russelii* had a t_90_ value of ~55 min [27]. In the current study, fraction 8 from the Javanese *D. siamensis* and Thai *D. siamensis* venoms at 1 µM (equivalent to approximately 13 µg/mL) displayed t_90_ values of ~157 min and ~117 min, respectively. In addition to low potency, the apparent lack of effect of these toxins in humans may be due to different susceptibility of species-specific targets [9,10]. For example, previous in vivo studies in rabbits found that Thai *D. siamensis* venom caused an acute hypotensive effect, without clear neurotoxic manifestations, despite the presence of neurotoxic PLA_2_s [46]. This suggests that the PLA_2_ toxins in *D. siamensis* venom are likely to exert their toxic effects in combination with other venom components, such as SVMPs, to induce other toxicities in vivo such as coagulopathy and haematological effects [46].

There are previous examples where snake venoms have been shown to contain pre-synaptic neurotoxins but do not cause neurotoxicity in humans. The “brown snake paradox” refers to the lack of neurotoxicity in humans following *Pseudonaja textilis* (Australian Eastern Brown snake) envenoming, despite the presence of the pre-synaptic neurotoxin textilotoxin in the venom. This is likely due to the low abundance (5.7%) of the toxin in the venom, relatively weak potency (t_50_: ~180 min), along with differences in neurotoxin-receptor interactions between humans and animals [47]. Our current findings suggest that the absence of neurotoxicity in humans following *D. siamensis* envenoming is more likely due to species-specific differences, given the high abundance and potency of the toxins in the venoms studied. This could be due to the fact that these pre-synaptic neurotoxins are not as potent at the human neuromuscular junction due to differences in receptors across different species (i.e., rodent or avian versus human) [47].

The myotoxic activity of the *D. siamensis* venoms examined in the current study appears to be largely confined to one of the two main PLA_2_ toxins, i.e., fraction 8. Fraction 8 from each *D. siamensis* venom exhibited in vitro myotoxicity that was more potent than previously identified PLA_2_ myotoxins from viper venom. Myotoxicity was only observed by the PLA_2_ toxins U1-viperitoxin-Dr1a and U1-viperitoxin-Dr1b from Sri-Lankan *D. russelii* at a higher concentration (3 µM) [40] compared to either the Javanese or Thai fraction. Interestingly, the myotoxic effects of the combination of fractions from Javanese *D. siamensis* venom were almost identical to the effects of fraction 8 alone, but significantly less than the whole venom. Therefore, it is likely that there are other components with myotoxic activity in the venom. This is supported by the observation that the removal of both fractions 8 and 10 from Javanese *D. siamensis* venom only resulted in a partial loss of myotoxicity. It is possible that the removal of PLA_2_ fractions contributed to a reduced intermolecular synergism, and hence, reduced pharmacological effect [41]. In contrast, the removal of fractions 8 and 10 from Thai *D. siamensis* venom resulted in a total loss of myotoxic activity, suggesting that the two isolated fractions are the components responsible for in vitro myotoxicity. However, it is clear that the PLA_2_ toxins isolated from Javanese and Thai *D. siamensis* venoms exert more neurotoxic effects than myotoxic effects at the concentrations examined. This supports previous work indicating that the myotoxicity of Thai *D. siamensis* and *D. russelii* venoms in both in vitro preparations and envenomed patients is absent, or relatively mild [30,48]. While myotoxic envenoming is less of a concern for victims bitten by Thai *D. siamensis* [8,35], in Indonesia, envenoming by *D. siamensis* may be associated with a higher frequency of myotoxicity [12]. Unfortunately, given that there is limited information about the magnitude and clinical symptomology of *D. siamensis* bites in Indonesia [49], further studies are required to elucidate the differences between the clinical effects of these venoms. Future studies could focus on measuring biochemical markers of muscle damage (e.g., creatine kinase (CK), lactate dehydrogenase (LDH), and myoglobin) to confirm myotoxicity in other animal models. By measuring these biomarkers, we could more accurately quantify the extent and nature of myotoxicity caused by *D. siamensis* venoms. This would provide a deeper understanding of the mechanisms underlying venom-induced muscle damage, assisting in the identification of more effective treatments and improving clinical outcomes for snakebite victims.

The pre-addition of Thai Russell’s viper monovalent antivenom significantly inhibited the in vitro pre-synaptic neurotoxic effects of fraction 8 from both venoms, indicating that the antibodies raised against the venom from the Thai species can also neutralise the key toxins in the Javanese venom. Previous research has demonstrated that Thai Russell’s viper monovalent can neutralise the neurotoxicity of whole venom from both Thai and Javanese *D. siamensis* [19], and has also shown neutralising capabilities against other Asian viper venoms, including Sri Lankan *D. russelii* [50]. The PLA_2_ inhibitor, Varespladib, also abolished the pre-synaptic neurotoxic effects of fraction 8 from both venoms, as well as the effects of the combination of fractions 8 and 10 from both venoms. Varespladib has previously been shown to prevent the inhibition of indirect twitches, i.e., neurotoxicity by crotoxin, a trimeric pre-synaptic neurotoxin, in the mouse neuromuscular preparation [51]. Of particular interest, in our study, was the ability of Varespladib to partially reverse the pre-synaptic neurotoxic effects of the fractions. Crystallographic and bioinformatic studies had revealed that Varespladib may disrupt the ability of toxins to target membranes or block docking sites for toxins on cell membranes [52]. Additionally, Varespladib was shown to prevent interactions between toxins by disrupting intra- and intermolecular interactions between two components and disrupt toxic activity [53]. In our previous study, Varespladib added 60 min after venom partially reversed the indirect twitch inhibition caused by Thai *D. siamensis* and Javanese *D. siamensis* whole venoms [19] to a similar level observed in the current study (i.e., ~50–60%). The reasons for a lack of full recovery of twitches are unclear. Considering that the main mechanisms of pre-synaptic neurotoxins in inducing neuromuscular inhibition is the depletion of neurotransmitter release [54], it is possible that resynthesis of neurotransmitters is limited in an isolated, in vitro preparation. Another possibility is that these pre-synaptic neurotoxins may interfere with physiological processes that Varespladib cannot reverse, as this agent primarily functions to inhibit PLA_2_-mediated toxicity, and not necessarily the impairment of physiological structures. For example, previous observations of the effects of β-bungarotoxin on mouse and rat phrenic nerve diaphragms showed irreversible processes such as the swelling of mitochondria that can affect motor nerve terminal function [55] and would not be immediately reversed by treatment. As hypothesised by Oliveira and colleagues in their reversal studies using *O. scutellatus* venom [56], the mechanisms of action of Varespladib could range from the blockage of target sites or disruption of internal and toxic mechanisms to removing PLA_2_ toxins. This could suggest that there must be an additional mechanism of action of PLA_2_-induced toxicity that the inhibitor may not target.

## 4. Conclusions

In conclusion, we have shown that both Javanese and Thai *D. siamensis* venoms contain two major PLA_2_ toxins that have in vitro pre-synaptic neurotoxic and/or myotoxic effects. Additionally, we have shown the pan-inhibitory effect of Varespladib against PLA_2_ toxins from different *Daboia* venoms, highlighting that Varespladib may be a valuable treatment against pre-synaptic neurotoxins from different snakes. These findings underscore the importance of understanding venom composition and interactions for developing more effective antivenom therapies and improving clinical outcomes for envenomed patients.

## 5. Materials and Methods

### 5.1. Animals

Male brown chicks aged five to ten days (White Leghorn crossed with New Hampshire) were purchased from Wagner’s Poultry in Coldstream, Victoria, Australia. The animals were housed at Monash Animal Facilities and provided with free access to food and water.

### 5.2. Chemicals and Drugs

The following drugs/chemicals were obtained from Sigma-Aldrich (St. Louis, MO, USA): acetylcholine chloride, bovine serum albumin (BSA), carbamylcholine, dimethyl sulfoxide (DMSO), potassium chloride, d-tubocurarine, trifluoroacetic acid (TFA), and Varespladib (CAS: 172732-68-2). Acetonitrile (ACN) was purchased from Merck (Darmstadt, Germany). All drugs were dissolved in Milli-Q water, except for Varespladib, which was initially dissolved to a final concentration of 1 mM in DMSO.

### 5.3. Venoms and Antivenoms

Thai *D. siamensis* venom was obtained from the Queen Savaobha Memorial Institute (QSMI), Thailand. Javanese *D. siamensis* venom was a gift from Venom Supplies, Tanunda, Australia. All venoms were pooled from several adult snakes of both sexes (<20 specimens of both male and female), freeze-dried, and stored at −20 °C.

For use in the chick biventer cervicis nerve-muscle preparation, the venoms were dissolved in 0.05% BSA and stored at 4 °C prior to use. Thai *D. siamensis* monovalent antivenom (LOT no. WR00221, MFG: 28/09/2021, Exp: 28/09/2026) was purchased from QSMI, Bangkok, Thailand. The freeze-dried contents were reconstituted with the supplied solution from the manufacturer. The amount of antivenom required to neutralise venom was based on the neutralisation ratio stated by the manufacturer, where 1 mL of Thai *D. siamensis* monovalent antivenom neutralises 0.6 mg *D. siamensis* venom. The equivalent amount of antivenom required to neutralise the in vitro effects of the venom fractions was calculated based on the relative abundance of the fraction in the whole venom.

### 5.4. Isolation and Purification of Toxins

#### 5.4.1. Reverse Phase-High Performance Liquid Chromatography (RP-HPLC)

Freeze-dried *D. siamensis* venom (2 mg) was reconstituted in 500 µL milli-Q water (Millipore Corporation, Billerica, MA, USA) to a final concentration of 4 mg/mL and centrifuged at 10,000 rpm for 10 min before being loaded into a Phenomenex Jupiter semi-preparative C18 column (5 µm, 250 mm × 10 mm; Phenomenex, Torrance, CA, USA) equilibrated with solvent A (0.1% TFA in milli-Q water) at a flow rate of 2 mL/min. Fractions were eluted using a gradient of solvent B (90% ACN and 0.09% TFA); 0–20% over 10 min, 20–80% between 10 and 60 min and 80–0% between 60 and 65 min at a flow rate of 2 mL/min. Eluent was monitored at 214 nm. Fractions were manually collected and lyophilised. Fractions were screened to identify those with neurotoxicity/myotoxicity using the chick biventer cervicis nerve-muscle preparation (see Section 5.5).

#### 5.4.2. Liquid-Chromatography Electro-Spray Ionisation Intact Protein Analysis Coupled with Mass Spectrometry/Mass Spectrometry (ESI-LCMS/MS)

Protein samples (1–3 µg) were analysed by LC-MS using a quadrupole TOF mass spectrometer (MicroTOFq, Bruker Daltonics, Bremen, Germany) coupled online with a 1200 series capillary HPLC (Agilent Technologies, Santa Clara, CA, USA). Samples were injected onto a MabPac SEC-1 5 µm 300A 50 × 4 mm (Thermo Scientific, Bremen, Germany) column with 50% ACN, 0.05% TFA, and 0.05% formic acid at a flow rate of 50 µL/min. The proteins were eluted over a 20 min runtime monitored by UV detection at 254 nm. The eluent was nebulised and ionised using the Bruker electrospray source with a capillary voltage of 4500 V dry gas at 180 °C, flow rate of 4 L/min, and nebuliser gas pressure at 300 mbar. After 20 min, the flow path was switched to infuse Low Concentration Tuning Mix (Agilent Technologies, Santa Clara, CA, USA) to calibrate the spectrum post acquisition. The spectra were extracted and deconvoluted using Data explorer software version 3.4 build 192 (Bruker Daltonics, Bremen, Germany).

#### 5.4.3. Intact Mass Spectrometry QTOF

Samples were analysed by LC-MS using a quadrupole TOF mass spectrometer ImpactII (Bruker Daltonics, Bremen, Germany) coupled online with an Elute UHPLC (Bruker Daltonics, Bremen, Germany). Samples were injected onto a MabPac SEC-1 4 × 150 mm column (Thermo Scientific, Bremen, Germany) with an isocratic mix of 40% buffer A (2% ACN, 0.1% Formic acid, 0.1% TFA) and 60% buffer B (80% ACN, 0.1% formic acid) at a flow rate of 100µL/min. The proteins were eluted over a 15 min runtime. The eluent was nebulised and ionised using the Bruker electrospray source with a capillary voltage of 4500 V, dry gas at 200 °C, flow rate of 8 L/min, and nebuliser gas pressure at 1.8 bar. Prior to analysis, the qTOF mass spectrometer was calibrated using ESI-L Low Concentration Tuning Mix (Agilent technologies, Santa, Clara, CA, USA). The spectra were extracted and deconvoluted using Data Analysis software version 5.1 (Bruker Daltonics, Bremen, Germany).

### 5.5. Chick Biventer Cervicis Nerve-Muscle Preparation

Male brown chicks were euthanised with CO_2_ inhalation. Two biventer muscles were dissected from each chick and subsequently mounted vertically onto wire holders under 1 g resting tension in a 5 mL organ bath. Tissues were maintained at 34 °C in physiological solution (composition: 118.4 mM of NaCl, 25 mM of NaHCO_3_, 11.1 mM of glucose, 4.7 mM KCl, 1.2 mM of MgSO_4_, 1.2 mM of KH_2_PO_4_, and 2.5 mM CaCl_2_), aerated with carbogen (95% O_2_ and 5% CO_2_). To evoke indirect twitches (i.e., neurotoxicity experiments), the motor nerves of the preparations were stimulated with a supramaximal voltage of 10–15 V at a frequently of 0.1 Hz with a duration of 0.2 ms using an LE series electrical stimulator. The twitches were recorded using a PowerLab system (ADInstruments Pty Ltd., Bella Vista, Australia) via a Grass FT03 force transducer. Tissues were allowed to equilibrate for over a period of 20 min. To test for selective nerve stimulation, dTC (10 µM) was added to the bath to ensure that twitches were abolished. The dTC was then washed from the bath using physiological solution until indirect twitches were restored. In the absence of electrical stimulation, contractile responses to exogenous ACh (1 mM, 30 s), CCh (20 µM, 60 s), and KCl (40 mM, 30 s) were obtained, with washing between each drug addition. Following this, electrical stimulation was recommended for at least 20–30 min until a steady twitch height was achieved. For direct twitches (i.e., myotoxicity experiments), the electrode was placed around the belly of the muscle, which was stimulated with a supramaximal voltage at a frequency of 0.1 Hz and a duration of 2 ms. Nerve stimulation was abolished by the addition of 10 µM dTC, which remained in the bath for the duration of the experiment.

The neutralising effects of antivenom or Varespladib were evaluated using either a protection or reversal protocol. For protection experiments, antivenom was added to the organ bath 20 min before the addition of fraction(s), or the fraction(s) were pre-incubated with Varespladib for 15 min before addition to the bath. For reversal experiments, Varespladib was added 60 min after the addition of the fraction(s). At the conclusion of the experiment, responses to ACh, CCh, and KCl were readded in the absence stimulation.

### 5.6. PLA_2_ Activity

PLA_2_ activities of *D. siamensis* venoms, and isolated fractions, were determined using a PLA_2_ assay kit (Cayman Chemical, Ann Arbour, MI, USA). According to the manufacturer’s instructions, venom and fraction stock solutions (1 mg/mL) were serially diluted to reach a final concentration of 0.5 µg/mL. The pre-mixed solution of venom or fraction samples, assay buffer, and indicator DTNB [5.5′-dithio-bis-(2-nitrobenzoic acid)] were added to a 96-well plate in duplicates. Substrate solution diheptanoyl thio-PC was then added to each well, and the plate was read every 2 min at a wavelength of 414 nm for 22 min using a ClarioStar plate reader. Absorbance values were measured to calculate PLA_2_ activity, expressed as micromoles of phosphatidylcholine hydrolysed per min per mg of enzyme (µmol/min/mg) of each sample dilution. The values indicated were measured as the mean of triplicate wells. Bee venom PLA_2_ was used as a positive control.

### 5.7. Data Analysis and Statistics

Twitch responses of the chick biventer cervicis nerve-muscle preparation were measured every 4 min and expressed as a percentage of their height at the commencement of the experiment prior to the addition of venom/fractions. Contractile responses to ACh, CCh, and KCl obtained at the conclusion of the experiment were expressed as a percentage of their corresponding pre-venom/fraction response. For myotoxicity experiments, changes in baseline tension were measured every 10 min after venom/fraction addition. One-way analysis of variance (ANOVA) was performed for comparisons between venom/fractions and controls. Comparisons of contractile responses to agonists pre- and post-venom/fraction were performed using Student’s paired *t*-test. All ANOVAs were followed by a Bonferroni multiple comparison. All data are expressed as the mean ± standard error of the mean (SEM), where n is the number of tissues. For all statistical tests, *p* < 0.05 was considered statistically significant. All analyses were performed using GraphPad Prism 10.2.3 (GraphPad Software, San Diego, CA, USA).

## Data Availability

The raw data supporting the conclusions of this article will be made available by the authors on request.
